# Points to consider in designing and conducting astigmatism research in cataract surgery

**DOI:** 10.3389/fopht.2026.1789801

**Published:** 2026-02-23

**Authors:** Atsushi Kawahara

**Affiliations:** Yoshida Eye Hospital, Hakodate, Japan

**Keywords:** astigmatism, blinking, cataract surgery, dry eye disease, statistical analysis, tear film, tear film break-up time, vector analysis

## Introduction

The defining characteristic of cataracts, the leading cause of blindness and visual impairment worldwide, is clouding of the crystalline lens (1). They are a major cause of blindness in developing countries (2), and a 2020 survey reported that approximately 78 million people globally suffer from visual impairment due to cataracts (3). Risk factors for cataracts include age, hypertension, diabetes, smoking, and sleep disorders (4–6). Among these, age is the most significant risk factor, and its prevalence increases with age (7). Consequently, the demand for cataract surgery has been rising in recent years.

Astigmatism is a refractive error caused by uneven curvature of the cornea and crystalline lens, resulting in multiple focal points that blur vision at all distances (8). Symptoms of astigmatism include reduced visual acuity, glare, halos, difficulty with night driving, falls, and dependence on eyeglasses. While prevalence rates in the general population vary widely from 8% to 62%, the prevalence is particularly high among individuals aged 70 and older (9). Hypotheses regarding the causes and mechanisms of astigmatism have been proposed, including genetics, maternal age, parental history of myopia, extraocular muscle tension, eyelid pressure, electronic screen time, and air pollution (10, 11).

Both cataracts and astigmatism have high prevalence rates among the elderly, making it highly likely that astigmatism coexists in patients eligible for cataract surgery. A report (12) indicated that over one-third of patients had astigmatism exceeding 1 diopter (D). In the modern era, advances in diagnostic equipment, surgical techniques, and intraocular lenses (IOLs) have transformed cataract surgery from a procedure solely for restoring vision to one that also corrects refractive errors (13). For example, intraoperative corneal astigmatism correction via toric IOL implantation is now widely performed (14). Consequently, numerous studies on astigmatism correction have been published, aiming to further enhance surgical success.

It is widely understood that neutralizing corneal astigmatism during cataract surgery is effective. In practice, techniques such as toric IOL implantation, steep-axis clear corneal incision, and limbal relaxing incision are employed. I routinely review papers on the subject of this thesis, and I regret that many papers fail to properly establish research protocols for improving astigmatism correction accuracy due to insufficient understanding of astigmatism and the precautions involved in its measurement. This paper aims to present fundamental concepts and points of caution in astigmatism research within cataract surgery practice, enabling researchers who understand these to obtain correct research results.

## Statistical analysis of astigmatism

**Figure 1:**
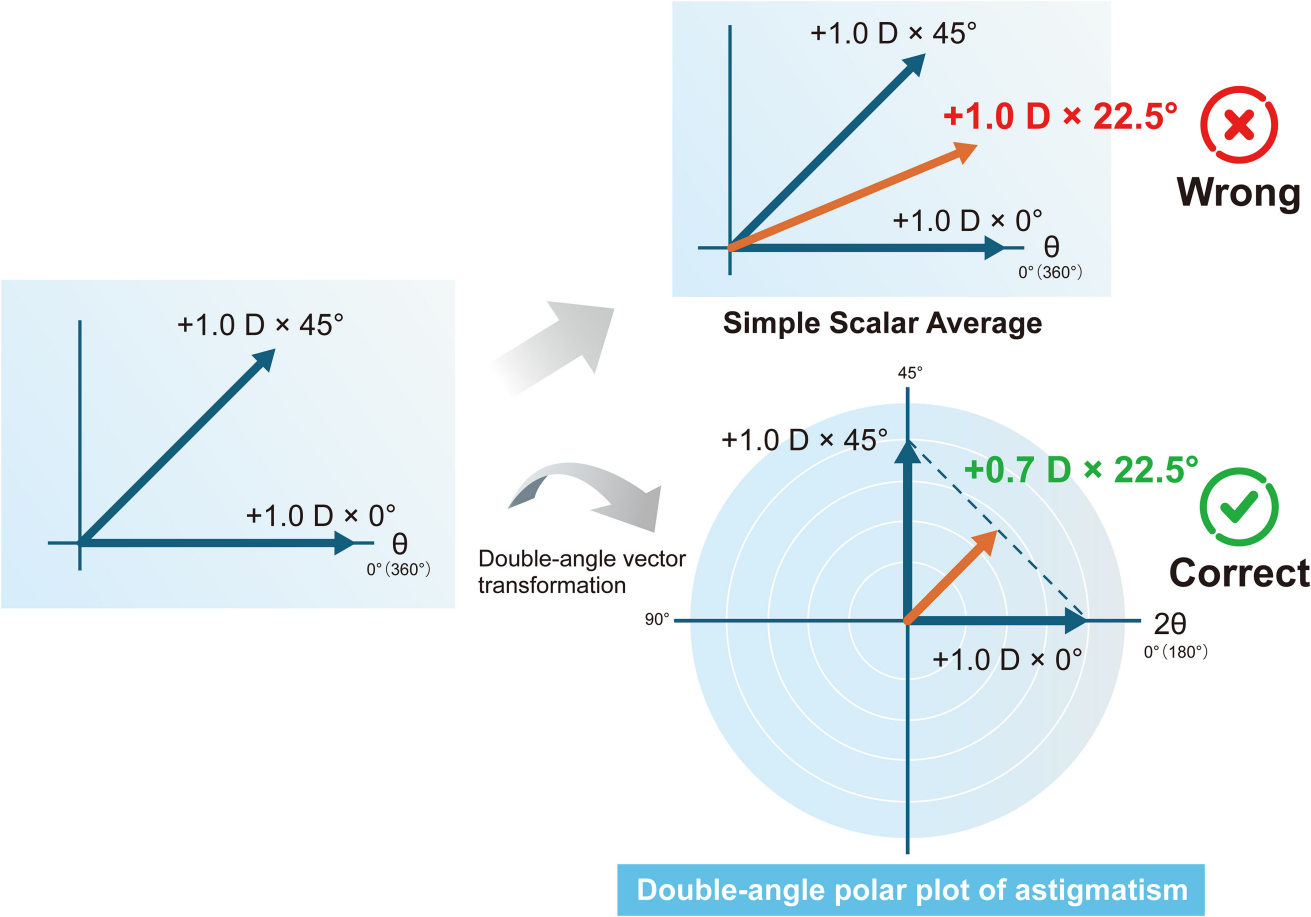
Mean of +1.0 D × 0° and +1.0 D × 45°.

**Figure 2:**
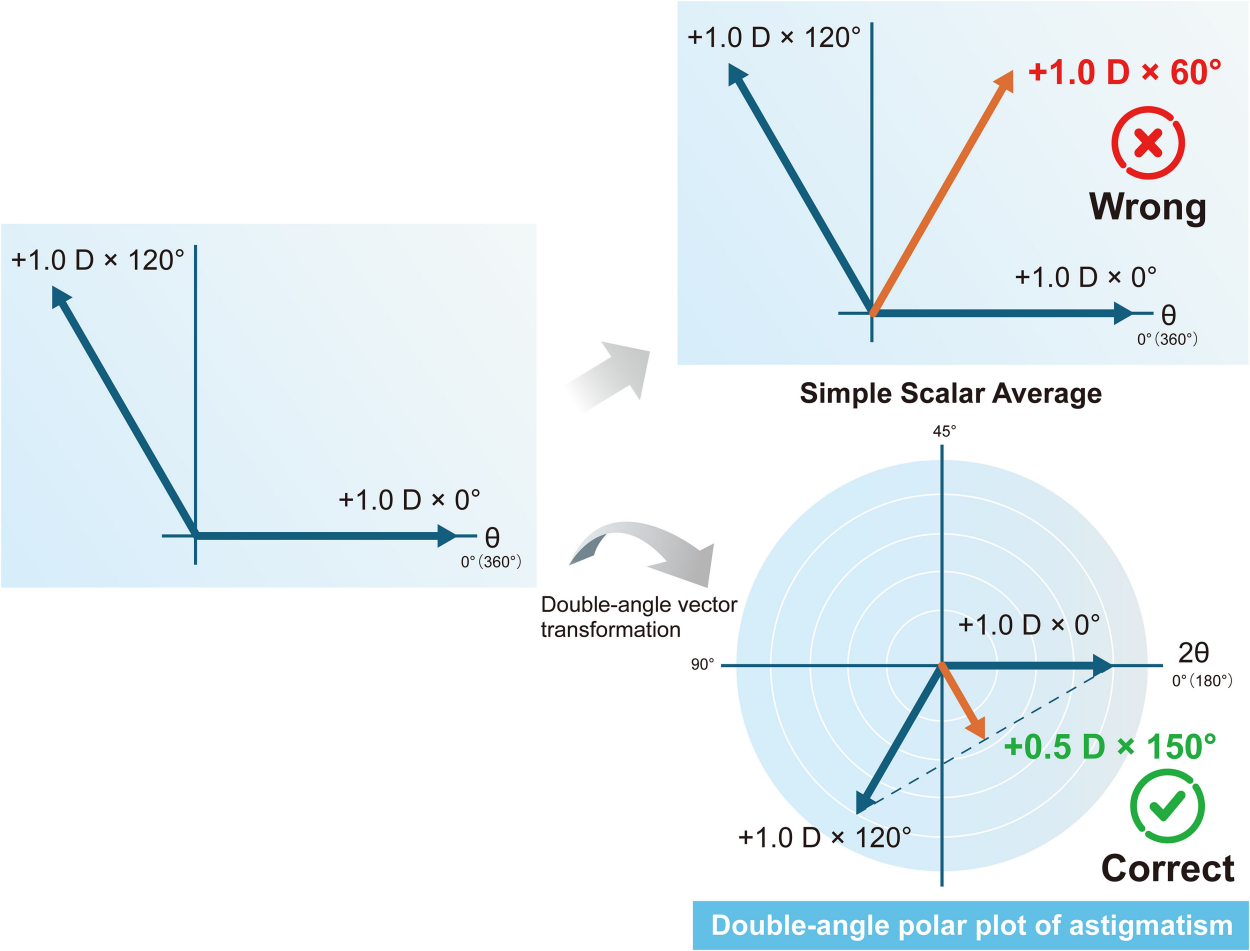
Mean of +1.0 D × 0° and +1.0 D × 120°.

Astigmatism is directional data. Directional data is data expressed as an angle relative to a reference direction. Other examples of directional data include earthquake hypocenter data in geology, wind direction data in meteorology, and optical axis data for crystals in physics. The application of statistical analysis to these types of data is radically different from handling non-directional data. Applying conventional statistical analysis to directional data risks leading to serious misinterpretations (15). Astigmatism is characterized as directional data with cylindrical refractive power and axis, making a vector the most appropriate representation method since it allows the combination of magnitude and direction to be expressed mathematically as a single entity. Astigmatism vector is generally denoted such as +1.0 D × 90°. In this way, since astigmatism is not a scalar quantity, there is little value in separating the power and axis and performing statistical analysis on each separately. Furthermore, astigmatism analysis possesses a characteristic unique to astigmatism: the refractive cylinder axis has to be doubled to find the vector axis (16). In astigmatism, the axis angle ranges from 0 to 180 degrees, whereas in geometry and trigonometry, angles range from 0 to 360 degrees. Therefore, to apply conventional geometry, trigonometry, and vector analysis to astigmatism, this discrepancy must be reconciled. This is why the astigmatism angle must be doubled. As a standard convention for plot transformation, polar coordinate plots must be performed for axes ranging from 0° to 180° (based on the axis before doubling) rather than from 0° to 360°. For example, the mean vector of +1.0 D × 0° and +1.0 D × 45° is +0.7 D × 22.5°, but if the mean values are calculated separately for the power and axis, the result becomes +1.0 D × 22.5°. Similarly, if the mean vector of +1.0 D × 0° and +1.0 D × 120° is calculated separately for the power and axis, it becomes +1.0 D × 60°, but the true mean vector is +0.5 D × 150° (**Figures 1**, **2**). Therefore, before performing statistical analysis on the measured astigmatism data, it is recommended to apply either the power vector method (15) or the X-Y coordinate analysis method (17) to the astigmatism vectors, which represent astigmatism as rectangular vector form. This allows each vector com-ponent to be treated as a scalar quantity (for standard statistical analysis). The above principles must also be considered when using the Alpins method (18), a representative vector analysis technique. The same applies to the surgically induced astigmatism vector, target-induced astigmatism vector, difference vector included in the Alpins method, and the metrics calculated using these. When performing statistical analysis on these, the aforementioned principles must be adhered to.

As in other fields, researchers studying astigmatism must strive to minimize bias as much as possible when collecting data for their research subjects. First, researchers conduct power analysis to determine the appropriate sample size for their study and create a protocol accordingly. Data is then collected following this protocol, and it is recommended that only one eye per case be included. This is because including data from both eyes of the same patient introduces bias due to interocular correlation, increasing Type I error (19). Furthermore, these data should be limited to the right eye only (or the left eye only). However, if for some reason the right eye and left eye data need to be mixed together, data conversion becomes necessary. This is because when averaging right and left eye data, the tendency for errors caused by cyclotorsional or antisymmetric healing patterns to cancel each other out must be eliminated (20). If the right eye is used as the reference for statistical analysis of astigmatism, the left eye astigmatism data must be mirrored horizontally to retain the correct nasal/temporal orientation with the right eye. Specifically, the astigmatism axis angle for the left eye must be converted using the following formula before being added as astigmatism data: Converted left eye axis = 180° – initial left eye axis (21). Finally, in studies requiring the handling of astigmatism data on the corneal plane (such as studies on toric IOLs), when using subjective refractive astigmatism data, the data must be converted from the spectacle plane to the corneal plane. For this calculation, reviewing the paper by Holladay et al. (22) is recommended.

## Astigmatism measurement considering tear film

The tear film on the cornea occupies the outermost layer of the eye and serves as the first refractive surface contacted by light entering the eye. Its optically significant role involves adjusting aberrations and refractive index that affect visual quality (23). When the tear film stability decreases and disorder occurs, those fluctuations distort the image projected onto the retina (24). Therefore, it is valid that astigmatism, a lower-order aberration, is influenced by the tear film, and the stable tear film is a necessary condition for accurate astigmatism measurement. The stability of the tear film has recently been primarily indicated by tear film break-up time (TBUT), and the increasing prevalence of short TBUT-type dry eye disease, characterized by reduced stability, has become a significant concern (25). Diagnosis of dry eye disease is recommended using the diagnostic criteria proposed by the Tear Film and Ocular Surface Society Dry Eye Workshop II (TFOS DEWS II), which is the current global standard (26). Diagnosis of dry eye disease requires subjective symptoms, but these are not particularly important in astigmatism measurement. Instead, objective clinical signs, especially shortened TBUT, are problematic. A systematic review (27) reported that shortened TBUT negatively impacts the measurement of corneal astigmatism. Rochct et al. reported that the astigmatism error after toric IOL implantation in the shortened TBUT group was 0.47 D, higher than the 0.37 D in the control group, with a significant difference between the two groups (28). This paper also reported that many patients required step changes in the optimal cylinder power of toric IOLs (e.g., changing from T3 to T4) due to measurement errors, and that measurement errors in the astigmatism axis of 10 degrees or more were significantly associated with TBUT. For precise astigmatism measurements, the stable tear film is essential. Therefore, evaluating a patient’s TBUT is a critical factor for researchers conducting astigmatism studies when selecting subjects. If any potential subjects have shortened TBUT, their tear film stability must be improved before they can be included in the study. Reports exist investigating whether instilling artificial tears or similar agents immediately before astigmatism measurement is effective (28–30), but no consistent conclusion has been reached. Therefore, these methods cannot be recommended at present. Consequently, when including patients with shortened TBUT in research, it is advisable to administer dry eye disease treatment for approximately 1 month before performing astigmatism measurement on these patients.

The tear film thickness is approximately 3–10 μm (31). Blinking causes the tear film to thin along the corneal surface due to tear evaporation (32, 33). If this thinning occurs uniformly in the optical zone, the effect of blinking on astigmatism would be negligible. However, changes in tear film-related astigmatism occur after blinking (34). After blinking, tear fluid evaporates and drains while flowing, causing its components to mix and separate. This results in the tear film’s thickness in the optical region having an orthogonal gradient, potentially allowing the tear film to function like an astigmatism lens. Indeed, the post-blink tear film exhibits uneven thinning, with bubbles and ridges observable (35, 36). Consequently, while dynamic equilibrium exists, the tear film remains optically unstable. Mrukwa Kominek et al. investigated the astigmatism formed in the tear film from immediately after blinking up to 15 seconds (37). Their report revealed that astigmatism formation within 5 seconds after blinking was small, increasing thereafter. This phenomenon was more pronounced in elderly patients and those with dry eye disease. Therefore, researchers studying astigmatism must be mindful that ocular biometry measurements for cataract patients should be completed within 5 seconds after blinking and opening the eyelids. This requirement must be incorporated into the research protocol.

## Conclusions

Cataract surgery has evolved into refractive surgery to a certain extent. Consequently, the precision of refractive data handled in research and the rigor of their analysis have become increasingly important in recent years. This paper emphasizes points to note in handling and measuring data in astigmatism research during cataract surgery, which researchers often overlook. First, it is essential to understand that astigmatism is a vector quantity and requires specific statistical analysis methods. Second, cataract surgeons must recognize that the tear film layer—the first refractive surface light encounters upon entering the eye—influences astigmatism. This understanding is crucial for selecting study subjects and measuring data. Finally, clinical research must be conducted by creating and adhering to a study protocol that incorporates the items presented in this paper. As a result, the accumulation of valuable research findings could open new avenues toward greater success in astigmatism correction during cataract surgery in the future.
